# Deaths caused by injury among people of working age (18–64) are decreasing, while those among older people (64+) are increasing

**DOI:** 10.1007/s00068-017-0827-1

**Published:** 2017-08-20

**Authors:** D. Bäckström, R. Larsen, I. Steinvall, M. Fredrikson, R. Gedeborg, F. Sjöberg

**Affiliations:** 1 Department of Anaesthesiology and Intensive Care, Vrinnevisjukhuset, Gamla Övägen 25, 603 79 Norrköping, Sweden; 2 Department of Anaesthesiology and Intensive Care, Universitetssjukhuset i Linköping, Linköping, Sweden; 3 Department of Clinical and Experimental Medicine, Linköping University, Linköping, Sweden; 4 Department of Hand Surgery, Plastic Surgery, and Burns, Linköping University, Linköping, Sweden; 5 Department of Surgical Sciences, Anaesthesiology and Intensive Care, Uppsala University, Uppsala, Sweden

**Keywords:** Injury, Trauma, Mortality, Working age, Elderly, Prehospital

## Abstract

**Background:**

Injury is an important cause of death in all age groups worldwide, and contributes to many losses of human and economic resources. Currently, we know a few data about mortality from injury, particularly among the working population. The aim of the present study was to examine death from injury over a period of 14 years (1999–2012) using the Swedish Cause of Death Registry (CDR) and the National Patient Registry, which have complete national coverage.

**Method:**

CDR was used to identify injury-related deaths among adults (18 years or over) during the years 1999–2012. ICD-10 diagnoses from V01 to X39 were included. The significance of changes over time was analyzed by linear regression.

**Results:**

The incidence of prehospital death decreased significantly (coefficient −0.22, *r* ^2^ = 0.30; *p* = 0.041) during the study period, while that of deaths in hospital increased significantly (coefficient 0.20, *r* ^2^ = 0.75; *p* < 0.001). Mortality/100,000 person-years in the working age group (18–64 years) decreased significantly (coefficient −0.40, *r* ^2^ = 0.37; *p* = 0.020), mainly as a result of decrease in traffic-related deaths (coefficient −0.34, *r* ^2^ = 0.85; *p* < 0.001). The incidence of deaths from injury among elderly (65 years and older) patients increased because of the increase in falls (coefficient 1.71, *r* ^2^ = 0.84; *p* < 0.001) and poisoning (coefficient 0.13, *r* ^2^ = 0.69; *p* < 0.001).

**Conclusion:**

The epidemiology of injury in Sweden has changed during recent years in that mortality from injury has declined in the working age group and increased among those people 64 years old and over.

## Introduction

Injury is a common cause of death in all age groups worldwide, and in the European Union more than 5.7 million people are treated in hospital each year [1]. In Sweden, 7% of all deaths are caused by injuries [1] and it has been estimated that 35% of the unintentional injuries in the working age group (18–64) could potentially be reduced [2]. The mechanism of injury in Scandinavia is usually blunt, and there are many deaths before patients reach hospital [3–5] because so many injuries happen in rural areas where reporting of the incident is delayed, journeys are longer, and mechanisms of injury are more dangerous [6, 7].

Changes in the number of deaths from injury over time can indicate whether preventive measures have been successful. Road traffic collisions are responsible for many deaths worldwide, and though there has been a decline in traffic collisions in Sweden over a long period, since 2002 this seems to have leveled off [8]. Self-harm is another common mechanism of injury [9].

Deaths after injury among the older age group (64+) is a growing problem among the increasingly aging population in western countries [10–13], and several authors have discussed this [10–12, 14, 15] as its burden on healthcare systems is increasing [10, 11], and older the patient the more likely they are to die from an injury [14, 15]. Mortality after injuries among children has also been reported as having declined [16, 17]. Few papers have focussed on people of working age, although they are most likely to be injured, [14, 15] and loss to the society from lost production is considerable. When we study the epidemiology of injuries we are faced with problems of recording, as most investigations rely on hospital data that do not fully cover the areas studied or the country investigated. Sweden is unique in epidemiological records with almost complete coverage of the country has been financed by the government in the form of the Cause of Death and National Patient Registries, and these are valid for epidemiological studies particularly as ICD-10 diagnoses of injury and causes of injury are included [9].

The aim of this study was to estimate the trends in mortality after injury over time for different mechanisms of injury.

## Methods

We studied all deaths from injury in Sweden during the 14-year period 1 January 1999–31 December 2012.

Sweden is mainly a rural country in northern Europe, and in 2012 had 9600,000 inhabitants. The emergency medicine service (EMS) that takes injured people to hospital is based on ambulances staffed with two paramedics, one of whom is a nurse. It is financed by the county councils and the service is similar across the country. The Prehospital Trauma Life Support (PHTLS) and the Advanced Trauma Life Support (ATLS) systems are used nationwide. Patients are usually taken to the nearest hospital and then, if necessary, transferred to a specialized trauma center, usually a tertiary referral center.

The cause of death of every Swedish citizen is included in the Swedish Cause of Death Registry. We identified those whose underlying cause of death was recorded as International Classification of Diseases, 10th Revision (ICD-10) diagnosis from V01 to X39, and refer to the underlying cause of death as the “mechanism of injury”. Admissions to hospital were gathered from the National Patient Registry. Death prehospital was defined as death with no registered hospital admission and death in hospital was defined as death with an admission date. We have included only the primary diagnosis as the cause of death; this excludes the late deaths caused by injury as they will not have an injury ICD code as a primary diagnosis.

Population data for the country were obtained from the Swedish Population Registry [18], and this was used for calculating mortality after injury as deaths/100,000 person-years. Incidence was calculated with age-specific populations.

The study was approved by the Regional Ethics Review Board in Linköping.

Data were analyzed with the help of STATISTICA version 12 (Dell Inc, Tulsa, OK, USA), and are presented as mean (SD) unless otherwise stated. The significance of differences between groups was assessed using the Chi-square and *t* tests, as appropriate, and associations were analyzed with linear regression. Probabilities of less than 0.05 were accepted as significant.

## Results

**Fig. 1:**
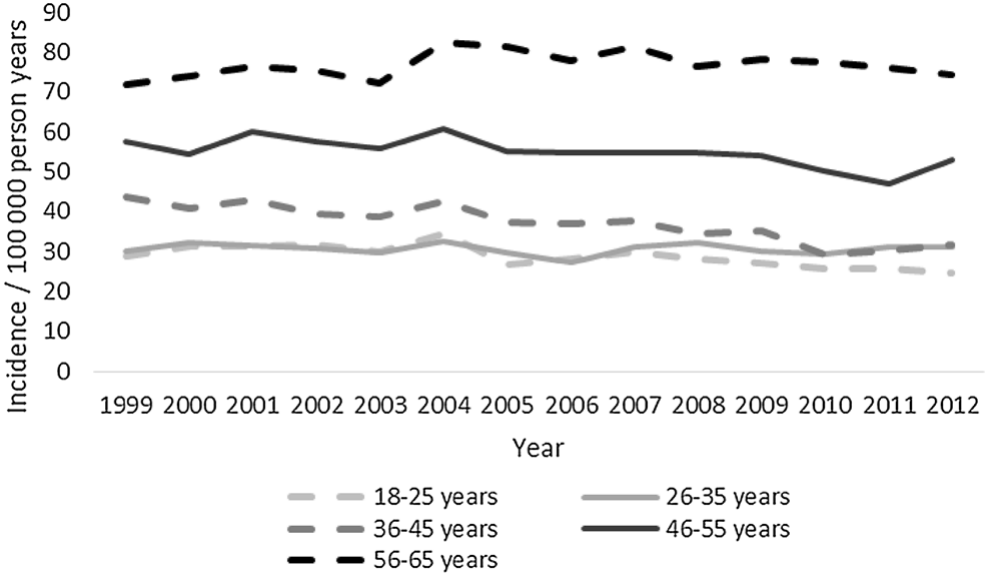
Mortality/100,000 in age groups 0–65 years

**Fig. 2:**
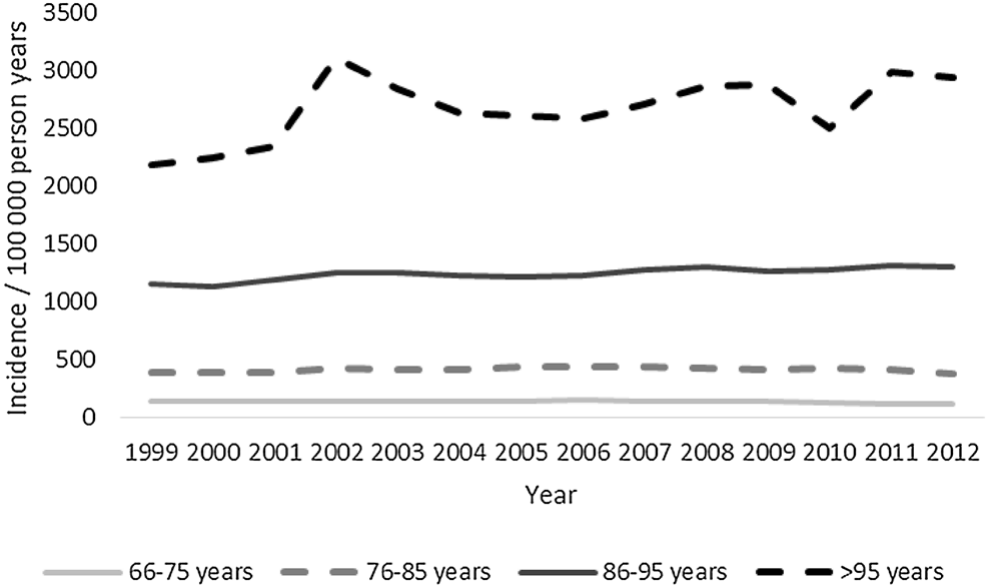
Mortality/100,000 in age groups 66 years and older

**Fig. 3:**
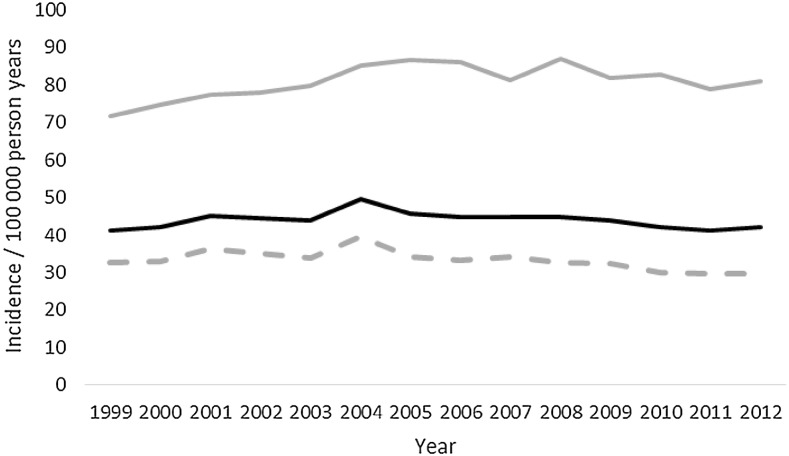
Mortality/100,000 by year. *Black* all, *gray* elderly, *dotted gray* working age

A total of 43,720 deaths were included in the study. Almost three-quarters died before admission to hospital, mean (range) age was 59 (18–109) years, almost a third were women, and almost two-thirds were between 18 and 64 years old (Table 1). Mortality increased with each age group except between the ages of 15–25 and 26–35 years (Figs. 1, 2). The most common overall mechanism of injury was self-harm, followed by falls and traffic collisions (Table 2). The overall mortality remained unchanged between 1999 and 2012, with 32 deaths/100,000 person-years at the start and 33 deaths/100,000 person-years in 2012 (Fig. 3; Table 3). Death caused by road traffic collisions, drowning, suffocation, self-harm, and assault declined during the period, whereas those caused by poisoning and falls increased (Table 3). The patients were divided into subgroups by age: those of working age (18–64 years), those who were older (64 and over), and whether the death was before admission or in hospital.

**Table 1:** Details of the patients

	Total (*n* = 43,720)	Working age (18–64 years) (*n* = 25,388)	Those over 64 years old (*n* = 18,332)	*p* value
Male	29,781 (68.1)	19,006 (74.9)	10,775 (58.8)	<0.001
Female	13,939 (31.9)	6382 (25.1)	7557 (41.2)	
Death before admission	31,829 (72.8)	22,211 (87.5)	9618 (52.5)	<0.001
Death in hospital	11,891 (27.2)	3177 (12.5)	8714 (47.5)	
Mean (SD) age (years)	58.8 (21.7)	45.4 (13.5)	82.9 (8.2)	<0.001

Data are number (%) unless otherwise stated. Working age and elderly compared by sex and deaths before and after admission by Chi-square, and in total by *t* test

**Table 2:** Distribution of deaths by mechanism of injury

ICD 10 code	All	Working age 18–64 years	Those aged over 64 years	*p* value
All	43,720 (100)	25,388 (100)	18,332 (100)	
Self-harm X60–X84	16,033 (36.7)	11,904 (47.0)	4129 (22.5)	<0.001
Fall W00–W19	10,319 (23.6)	1393 (5.5)	8926 (48.7)	<0.001
Vehicle, traffic V01–V99	6266 (14.3)	4539 (17.9)	1727 (9.4)	<0.001
Poisoning X40–X49	4315 (9.9)	3688 (14.5)	627 (3.4)	<0.001
Drowning W65–W74	1309 (3.0)	759 (3.0)	550 (3.0)	0.95
Suffocation W75–W84	1267 (2.9)	383 (1.5)	884 (4.8)	<0.001
Assault X85–Y09	1117 (2.6)	964 (3.8)	153 (0.8)	<0.001
Fire, smoke, hot objects X00–X19	1108 (2.5)	501 (2.0)	607 (3.3)	<0.001
Natural/environmental X20–X39	1092 (2.5)	661 (2.6)	431 (2.3)	0.10
Machinery W20–W64	776 (1.8)	498 (2.0)	278 (1.5)	<0.001
Electricity, radiation W85–W99	95 (0.2)	75 (0.3)	20 (0.1)	<0.001
Police, war Y35–Y36	23 (0.0)	23 (0.0)	0 (0.0)	<0.001

Data are number (%). Comparisons by Chi-square test

**Table 3:** Changes in incidence over time by men and women and mechanism of injury

	All	Men	Women
All			
Coefficient	−0.03	−0.19	0.12
*r* ^2^	0.00	0.12	0.10
*p* value	0.829	0.229	0.265
Self-harm			
Coefficient	−0.11	−0.14	−0.08
*r* ^2^	0.51	0.47	0.30
*p* value	0.004	0.007	0.043
Fall			
Coefficient	0.34	0.32	0.36
*r* ^2^	0.88	0.81	0.87
*p* value	<0.001	<0.001	<0.001
Vehicle, traffic			
Coefficient	−0.27	−0.41	−0.14
*r* ^2^	0.88	0.85	0.88
*p* value	<0.001	<0.001	<0.001
Poisoning			
Coefficient	0.17	0.27	0.07
*r* ^2^	0.84	0.80	0.69
*p* value	<0.001	<0.001	<0.001
Drowning			
Coefficient	−0.04	−0.08	−0.01
*r* ^2^	0.67	0.67	0.30
*p* value	<0.001	<0.001	0.041
Suffocation			
Coefficient	−0.02	−0.03	−0.01
*r* ^2^	0.55	0.48	0.11
*p* value	0.002	0.006	0.248
Assault			
Coefficient	−0.03	−0.04	−0.02
*r* ^2^	0.48	0.35	0.34
*p* value	0.006	0.026	0.028
Fire, smoke, hot objects			
Coefficient	−0.02	−0.02	−0.02
*r* ^2^	0.23	0.12	0.30
*p* value	0.081	0.218	0.042
Natural/environmental			
Coefficient	−0.04	−0.04	−0.03
*r* ^2^	0.01	0.02	0.01
*p* value	0.691	0.616	0.753
Machinery			
Coefficient	−0.01	−0.01	0.01
*r* ^2^	0.08	0.14	0.00
*p* value	0.341	0.20	0.91

Incidence over time has only been calculated in groups with more than 140 deaths. The association between incidence and year was analyzed with simple linear regression. The coefficient shows how much the incidence changed (mean value) each year

### Working age

Mortality among those of working age declined (coefficient −0.40, *r* ^2^ = 0.37; *p* = 0.02), and their most common mechanisms of injury were self-harm followed by road traffic collisions, and poisoning (Table 2). Simple linear regression showed a decline of 0.4/100,000 person-years (*p* = 0.02) and, after the model had been adjusted for age, the decline was 0.53/100,000 person-years (*p* = 0.002) and the incidence increased with 3.66/100,000 person-years for each year in age (*p* = 0.02).

Mortality declined for the following injuries: traffic collisions (coefficient −0.34, *r* ^2^ = 0.85; *p* < 0.001), drowning (coefficient −0.05, *r* ^2^ = 0.58; *p* = 0.002), and assault (coefficient −0.03, *r* ^2^ = 0.36; *p* = 0.022). The only mechanism of injury for which mortality had increased was poisoning (coefficient 0.23, *r* ^2^ = 0.78; *p* < 0.001). Mortality was reduced overall both in hospital (coefficient −0.09, *r* ^2^ = 0.60; *p* = 0.001) and before admission (coefficient −0.31, *r* ^2^ = 0.29; *p* = 0.048).

## 64 years old or more

Mortality increased in the group of elderly people during the study period (Fig. 3). Simple linear regression showed that for each year the mortality increased with 0.59/100,000 person-years (*p* = 0.046). After the model had been adjusted for age (the variable “age” was not significant, coefficient −3.6), the increase in incidence was 1.39/100,000 person-years (*p* = 0.03). As age was not significant in the regression, the increase in mortality does not seem to be an effect of an older population.

There were two mechanisms that increased: fall (coefficient 1.71, *r* ^2^ = 0.84; *p* < 0.001) and poisoning (coefficient 0.13, *r* ^2^ = 0.69; *p* < 0.001). The following declined: traffic (coefficient −0.41, *r* ^2^ = 0.82; *p* < 0.001), drowning (coefficient −0.10, *r* ^2^ = 0.50; *p* = 0.005), suffocation (coefficient −0.11, *r* ^2^ = 0.77; *p* < 0.001), fire, smoke, hot objects (coefficient −0.08, *r* ^2^ = 0.29; *p* = 0.049), self-harm (coefficient −0.45, *r* ^2^ = 0.75; *p* < 0.001), and assault (coefficient −0.05, *r* ^2^ = 0.50; *p* = 0.005). The most common mechanism of injury among elderly people was a fall, and it was more than twice as common as the second most common mechanism (self-harm), which was followed by traffic (Table 2). Mortality before admission to hospital decreased (coefficient −0.51, *r* ^2^ = 0.63; *p* = 0.001), and that after admission to hospital increased (coefficient 1.10, *r* ^2^ = 0.73; *p* < 0.001).

### Sex

**Fig. 4:**
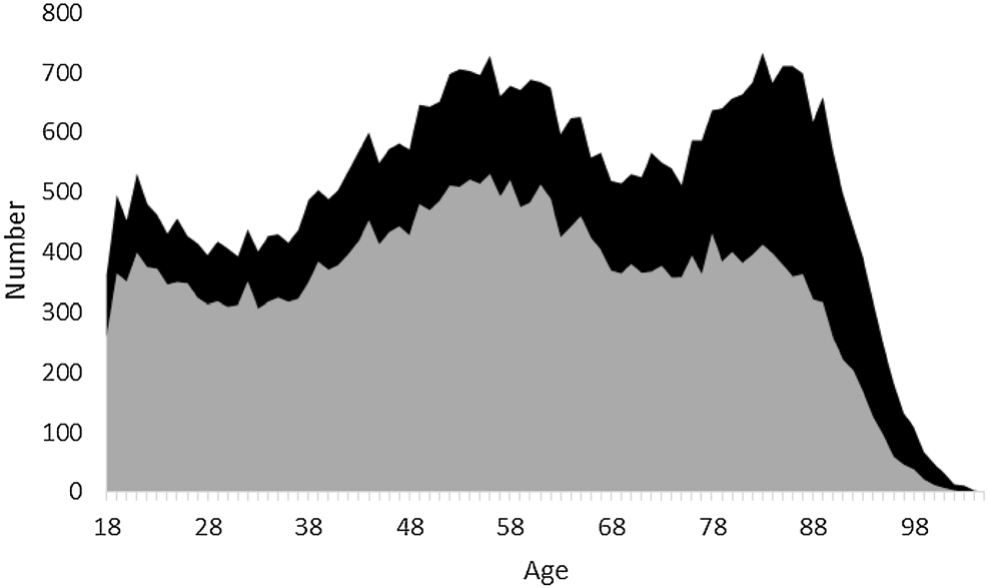
Crude mortality by age. *Black* women, *gray* men

More men died from injuries (Table 1) in all groups except for the oldest, as there are more women than men in the older group (Fig. 4). There was a difference in the distribution of men and women in the “working age” and “elderly” age groups, and this was more pronounced in the elderly age group than in the working age group, with more than 40% being women (Table 1). In the working age group mortality among men had declined (coefficient −0.54, *r* ^2^ = 0.63; *p* = 0.015), while there was no difference among the women. In the elderly, there was no difference over time among men, but mortality increased among elderly women (coefficient 1.06, *r* ^*2*^ = 0.70; *p* < 0.001) (Fig. 3). When the model was adjusted for age, it was not found to be a significant variable in the sub-groups “elderly men” and “women”.

Mortality from falls and poisonings increased among both men and women (Table 3). Traffic collisions, drowning, self-harm, and assault declined in both men and women, and suffocation declined among men and fire, smoke, and hot objects declined among women (Table 3).

### Deaths before admission to hospital

More people died before admission among the working age group than among the elderly patients (Tables 1, 4). The mechanisms of injury with the most deaths before admission to hospital were electricity and radiation, followed by drowning, self-harm, and poisoning. In the working age group, nature/environment had the highest proportion followed by electricity and radiation, drowning, and poisoning. In the elderly, electricity and radiation was followed by drowning, self-harm, and assault.

**Table 4:** Distribution of the deaths before admission by mechanism of injury and percentage of all patients studied

ICD 10 codes	All	Working age	Those aged over 64 years	*p* value
All	31,829 (72.8)	22,211 (87.5)	9618 (52.5)	
Vehicle, traffic	4891 (78.1)	3840 (84.6)	1051 (60.9)	<0.001
Fall	3095 (30.0)	795 (57.1)	2300 (25.8)	<0.001
Machinery	589 (75.9)	420 (84.3)	169 (60.8)	0.42
Drowning	1234 (94.3)	715 (94.2)	519 (94.4)	<0.001
Suffocation	868 (68.5)	308 (80.4)	560 (63.3)	<0.001
Electricity, radiation	91 (95.8)	71 (94.7)	20 (100)	0.09
Fire, smoke, hot objects	872 (78.7)	446 (89.0)	426 (70.2)	<0.001
Natural/environmental	945 (86.5)	630 (95.3)	315 (73.1)	0.03
Poisoning	3864 (89.5)	3400 (92.2)	464 (74.0)	<0.001
Intentional self-harm	14,390 (89.8)	10,720 (90.1)	3670 (88.9)	<0.001
Assault	973 (87.1)	849 (88.1)	124 (81.0)	<0.001
Police, war	17 (73.9)	17 (73.9)	0 (0.0)	0.007

Data are number of deaths before admission and (percentage of all deaths before admission compared to all deaths with that injury and age-group (working age or those aged over 64 years). Chi-square test used to compare those of working age with those aged over 64 years

**Fig. 5:**
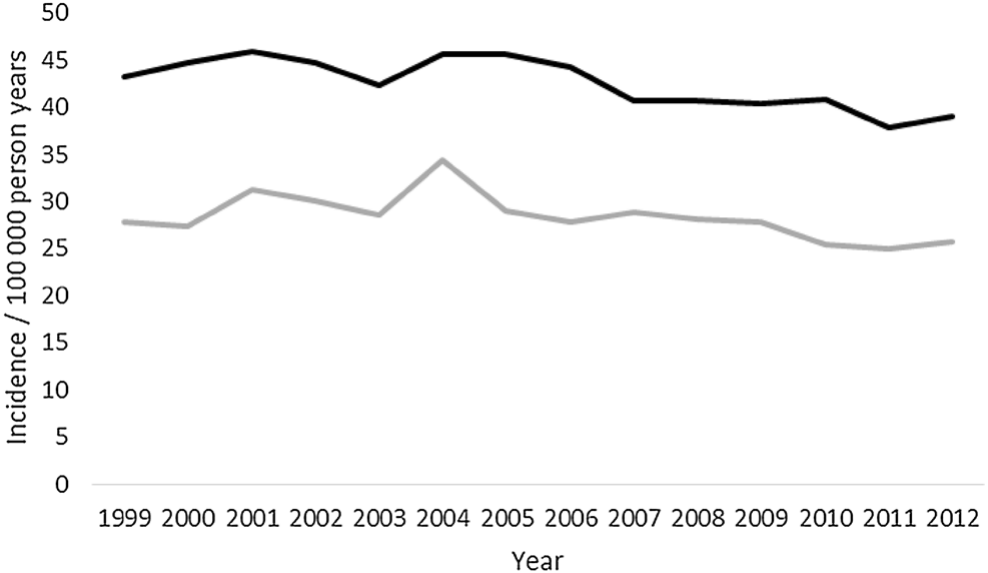
Mortality before admission/100,000 by year. *Black* aged 64 and over, *gray* working age

The incidence of death before admission decreased (coefficient −0.22, *r* ^2^ = 0.30; *p* = 0.041), while the number of deaths in hospital increased (coefficient 0.20, *r* ^2^ = 0.75; *p* < 0.001). Deaths before admission declined in both working age (coefficient −0.31, *r* ^2^ = 0.29; *p* = 0.048) and elderly people (coefficient −0.51, *r* ^2^ = 0.63; *p* = 0.001) (Fig. 5), but increased among those with falls (coefficient 0.05, *r* ^2^ = 0.53; *p* = 0.003) and poisoning (coefficient 0.16, *r* ^2^ = 0.84; *p* < 0.001), though they declined after traffic collisions (coefficient −0.22, *r* ^2^ = 0.87; *p* < 0.001), drowning (coefficient −0.04, *r* ^2^ = 0.64; *p* = 0.001), suffocation (coefficient −0.03, *r* ^2^ = 0.78; *p* < 0.001), and self-harm (coefficient −0.08, *r* ^2^ = 0.40; *p* = 0.015).

## Discussion

There was a decline in mortality among those of working age and an increase among those aged over 64, in contrast to the overall study group (Fig. 3). There was also a distinct difference in the mechanism of injury that led to death between those of working age and the older group (Table 2). Surprisingly, we found that mortality in hospital had increased, whereas others have reported a decrease [19, 20]. We think that falls may contribute to the rise in hospital mortality, as they were the most common injury in hospital, and the number increased during the time studied. As expected, mortality was higher among the older patients [14, 15].

We found a ratio of 2.1:1 (68% men, 32% women) which is similar to other reports from Scandinavia [3, 9, 21]. In both groups mortality peaked at the age of 55 years, and women seemed to have second peak at the age of 85 (Fig. 4). We have previously shown that the pattern of mortality after injury in children differs between boys and girls [17], which was also the case here, with a reduction in working age men. We also found an increase in mortality in elderly women, which was not a result of an increase in age in women. The changes in different mechanisms of injury over time showed a similar pattern between the sexes in almost all of the mechanism groups (Table 3).

The most common mechanism of injury among those of working age was self-harm, which was more than twice as common as injuries from vehicle and traffic collisions (Table 2) in the working age population. Kristiansen et al. [5]. also found it to be the most common cause of death from injury in the working age population, although not to the same extent. We have shown in a previous study during the same period that self-harm is a common mechanism of injury in children in Sweden [17]. Soreide et al. [3]. reported that suicide was responsible for 21% of (54 of 269) the deaths, and another Norwegian study by Bakke et al. [22] found it to be 33% (87 of 266), which is close to the 37% that we found in adults (Table 2). In the working age group, self-harm was responsible for almost half of the deaths (Table 2), but during the study period the incidence declined in both those of working age and the older group.

We think that the large numbers of self-inflicted injuries in part also explain the large numbers of deaths before admission among those of working age (Table 4). Suicide has a high incidence of death before admission [6, 22] because of the mechanism of injury, which is designed to be lethal. Injury-related deaths have been reported to be between 45 and 85% in Scandinavia [3–6, 23, 24]. Wisborg et al. [4] found the highest number with 85% (110 of 130) and Kristiansen et al. [5] (who studied a group of working age in Norway) found a 78% (6589 of 8466) overall mortality before admission. Our finding of 88% in the working age is higher, but our overall mortality outside hospital was only 72% (Table 1). When we looked at different injury mechanisms, our numbers were also higher. Self-harm was 39% (3337 of 8466) in the study by Kristiansen et al., whereas we found 90%, traffic collisions were 30% (2548 of 8466) in their study and 85% in ours, and falls were 8% (685 of 8466), whereas we found 57% (Table 4) [5].

There is no national organized system for emergency physicians to see patients before admission to hospital in Sweden. In some parts of the country there is an emergency doctor available for set hours, but during the study period that was rare. Some studies have suggested that emergency physicians available outside hospital have an impact on mortality from trauma [25, 26], and it may be that the lack of them in Sweden contributes to the large numbers of deaths in the community. No major changes have been made in the trauma system during the study period. These death rates are higher in rural areas [5, 6], which could also contribute to our findings as many Swedes live in rural areas.

The second most common cause of death among those of working age was injuries related to vehicle and traffic collisions. In 2002 the decrease in vehicle-related mortality seemed to have leveled off in Sweden [8], thus we did not expect to find a distinct decline during the whole period (Table 3). Traffic-related injuries have been reported to be the most common cause of death from injury in Scandinavia in several studies [3, 4, 21, 24]. Improvements in medical technology seem to have had an impact on this, and could be a contributory factor [27]. During the period there have been several preventive measures taken under the “zero policy” implemented by The Swedish Transport Administration. Our data support this as a good example of how preventive measures can affect mortality.

As many others have shown before us, the most common mechanism of injury among elderly patients was falls [11, 12, 14, 23, 28–30], though this is not a common cause of death from injury among those of working age (Table 2). Looking at the whole population, a fall seems to be an important mechanism of injury (Table 2) although it is obvious from our results that preventive measures should be focussed on elderly patients. We also found an increase in the incidence of falls among the older age group, which confirms the need for further preventive measures (Table 3).

We were surprised to find that poisoning was more common among those of working age than among those aged over 64 (Table 2), one reason for which could be that poisoning as a suicide attempt will be classified in this way when the intention is unknown.

One of the strengths of this study is that we used a government-financed database that applies to every Swedish inhabitant, and thus this gives us a complete picture without the risk of bias. The necropsy rate in this database was 79% of those who died of injuries before admission to hospital [9]. A limitation of the study is the retrospective design, which requires that the physicians have filled in the death certificates correctly. The long study period (14 years) is a strength, as it allows the development of the picture over time and lowers the risk of drawing conclusions from a single deviant year. When we studied those of working age, it was a disadvantage that we were not able to identify work-related injuries. The Swedish Cause of Death Registry uses ICD-10, and unfortunately it does not give us that information. A strength, though, was that as we included all deaths from injury we were able to show that most died before admission to hospital (Table 1), and that such deaths are decreasing over time. If they are excluded, important information about death from injury and the mechanisms involved would be lost, and conclusions would not be as solid. We think that our findings are important to enforce the preventive work that is being done in the society and in the organization of healthcare.

## Conclusions

Figures about the epidemiology of injury in Sweden have changed during recent years in that mortality from injury has declined among those of working age, but increased among people aged 64 and over. We also found a distinct difference in the mechanism of injuries that result in death between those of working age and those over 64.

There was a reduction in traffic-related injuries in among people of working age, and an increase in deaths after falls, mainly in the group over the age of 64.
